# Oxygen Reduction Reaction on PtCo Nanocatalyst: (Bi)sulfate Anion Poisoning

**DOI:** 10.1186/s11671-018-2574-6

**Published:** 2018-05-18

**Authors:** Jie Liu, Yan Huang

**Affiliations:** 1 Centre of Flexible and Printable Electronics, Harbin Institute of Technology (Shenzhen), Shenzhen, 518055 China; 2 School of Materials Science and Engineering, Harbin Institute of Technology (Shenzhen), Shenzhen, 518055 China; 3 State Key Laboratory of Advanced Welding and Joining, Harbin Institute of Technology (Shenzhen), Shenzhen, 518055 China

**Keywords:** (Bi)sulfate contamination, ORR kinetics, Transfer coefficient, Tafel slope, PtCo/C electrocatalyst

## Abstract

Pt alloy electrocatalysts are susceptible to anion adsorption in the working environment of fuel cells. In this work, the unavoidable bisulfate and sulfate ((bi)sulfate) poisoning of the oxygen reduction reaction (ORR) on a common PtCo nanocatalyst was studied by the rotating disk electrode (RDE) technique, for the first time to the best of our knowledge. The specific activity decreases linearly with the logarithm of (bi)sulfate concentration under various high potentials. This demonstrates that the (bi)sulfate adsorption does not affect the free energy of ORR activation at a given potential. Moreover, it is speculated that these two conditions, the adsorption of one O_2_ molecule onto two Pt sites and this adsorption as a rate-determining step of ORR reaction, are unlikely to exist simultaneously.

## Background

Pt alloy electrocatalysts have been demonstrated to be superior to Pt in polymer electrolyte membrane fuel cells (PEMFC) due to their higher activity toward oxygen reduction reaction (ORR) [1–16]. However, other considerations such as susceptibility to anion adsorption and surface oxide growth can affect the ORR behavior. The oxide can form in the presence of water according to the following reaction (and/or variants thereof):

1 $$ \mathrm{Pt}+{\mathrm{H}}_2\mathrm{O}=>\mathrm{PtOH}+{\mathrm{H}}^{+}+{\mathrm{e}}^{-} $$

Minute concentrations of various anions, such as (bi)sulfate and halides, always exist even in super-clean fuel cell systems. Both the formation of surface oxides and anion adsorption are potential dependent [17–19]. Most of the oxide can be generally removed by decreasing the potential to below 0.6 V vs. eversible hydrogen electrode (RHE), still within the cathode potential range of an operating fuel cell vehicle. The removal of anions may require potentials lower than those reached by an air-filled fuel cell cathode.

Anion adsorption on pure Pt single crystals and polycrystalline surfaces has been well documented [20–23]. Using a thermodynamic analysis, Herrero et al. [24] obtained a potential-dependent electrovalency of one to two electrons per adsorbed anion at high potentials, resulting from the competition between SO_4_^2−^ and HSO_4_^−^ adsorption on Pt(111). Kolics and Wieckowski [25] utilized a modified radioactive labeling method on Pt(111) and observed results consistent with those of Herrero [24]. Electrocapillary thermodynamics and modeling of H/OH adsorption in competition with SO_4_^2−^ adsorption was conducted by Garcia-Araez et al. [26–29]. In situ surface X-ray scattering showed diverse structures of halide anion adsorption arising from different strengths of Pt-halide interaction [30]. The rotating ring-disk electrode (RRDE) technique was applied to obtain bromide adsorption isotherms and to study the effects of bromide and sulfuric acid on ORR kinetics [7, 31–34]. All these studies were performed on continuous Pt layers or bulk Pt-Co alloy surfaces [7]. Anion adsorption on carbon-supported Pt and Pt alloy nanoparticles has been investigated by X-ray adsorption spectroscopy (XAS), discriminating between direct-contact and site-specific adsorption [35, 36]. Effects of chloride ions on the poisoning of the ORR on carbon-supported Pt nanoparticles have been reported [37], showing that the reduction of O_2_ to water was inhibited, while H_2_O_2_ production increased as the concentration of chloride in the electrolyte increased. Among the various anions, (bi)sulfate contamination is of key importance in PEMFCs due to a large presence of sulfonate groups, in perfluorosulfonate membrane/ionomer, which can be converted to free (bi)sulfate anions upon chemical degradation of the ionomer. Inspired by the work of Kabasawa et al. [38] who reported a linear relationship between the mass activity of Pt/C catalyst at a single potential (0.85 V) and the logarithm of the (bi)sulfate concentration in a single cell operated at 80 °C, we studied the effect of (bi)sulfate concentration on ORR activity of carbon-supported PtCo nanoparticles at multiple potentials. Polymer electrolyte membrane (PEM) fuel cells are of high technological importance for energy storage and transportation. However, Pt alloy nanocatalysts are unavoidable to (bi)sulfate adsorption because they are covered with sulfonated ionomers in PEM fuel cells. This paper is the first attempt to quantitatively measure the effect of sulfate adsorption on practical C-supported Pt alloy nanoparticles.

## Methods

### Materials

We used a common nanocatalyst of 30 wt.% PtCo supported on high-surface carbon (Tanaka Kikinzoku, Japan). A mixed 15 ml solution of ultrapure water (Milli-Q® system, Millipore, MA, USA), 2-propanol (HPLC grade, Sigma-Aldrich, USA), and a 5.37 wt% Nafion® solution (solvents: ethanol from Sigma-Aldrich, USA; water from Milli-Q® system, Millipore, MA, USA) with a volume ratio of 200:50:1, mixed with 15 mg of catalyst, was prepared and sonicated for 5 min. Ten microliters of ink was then transferred onto the glassy carbon surface with a geometric area of 0.196 cm^2^. The electrode was dried in air for 1 h before measurement.

### Evaluation of the Electrochemical Measurement

RDE measurements were conducted in a three-electrode electrochemical cell setup using a potentiostat, a rotation control (Pine Instrument Co, USA), and a 0.1-M HClO_4_ base electrolyte. A silver/silver chloride reference electrode was separated from the working electrode compartment by a salt bridge. All reported potentials refer to that of the reversible hydrogen electrode (RHE). H_2_SO_4_ (Veritas® doubly distilled, GFS Chemicals, OH, USA) solution was injected into the electrolyte to obtain the desired concentrations. Positive-going ORR curves starting from 0.05 V at 5 mV/s were obtained in an O_2_-saturated electrolyte under a rotation speed of 1600 rpm. All measurements were carried out at room temperature.

## Results and Discussion

**Fig. 1:**
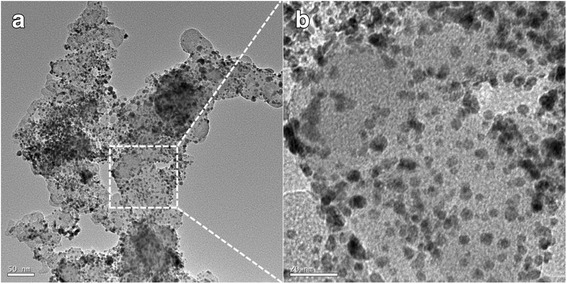
**a** TEM image of the nanocatalyst, scale bar = 50 nm. **b** Zoomed-up TEM image, scale bar = 20 nm

**Fig. 2:**
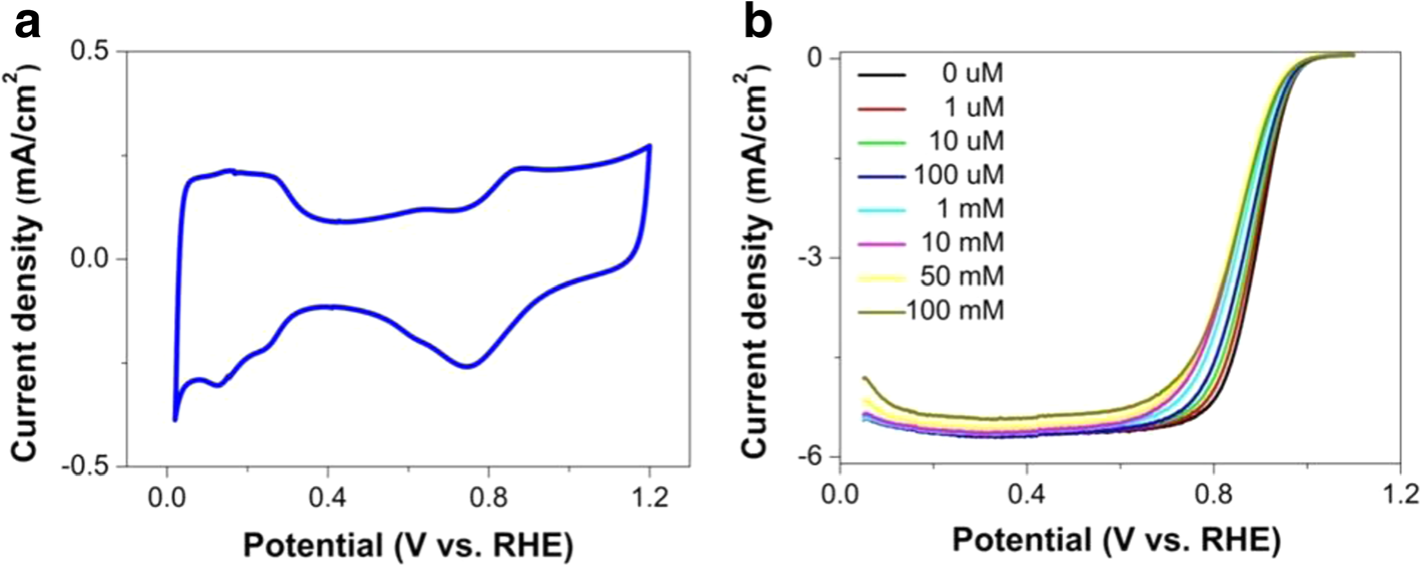
**a** CV curve at a sweep rate of 20 mV/s. **b** LSV curves at various (bi)sulfate ion concentration from 0 to 100 mM

As clearly shown in Fig. 1, the PtCo nanoparticles have a size ranging from 3 to 7 nm, and they are uniformly distributed on carbon. Positive-going and negative-going scans of the cyclic voltammetry (CV) profile are almost symmetrical with respect to the current density axis, indicating a reversible adsorption behavior (Fig. 2a). The CV curve shows features corresponding to the adsorption/desorption of hydrogen at around 0.15 V vs. RHE and oxidation/reduction of Pt at 0.85 V/0.79 V vs. RHE. We used 210 μC/cm^2^ Pt as the saturated H adsorption charge. Therefore, the surface area of the carbon-supported PtCo electrocatalyst is 62 m^2^/gPt. The linear sweep voltammetries (LSVs) show a clear dependence of the current density on the anion concentration as they shift toward negative potentials as the anion concentration increases (Fig. 2b). Both the half-wave potential and the diffusion current of the polarization curve shift, suggesting that there is apparent activity loss.

**Fig. 3:**
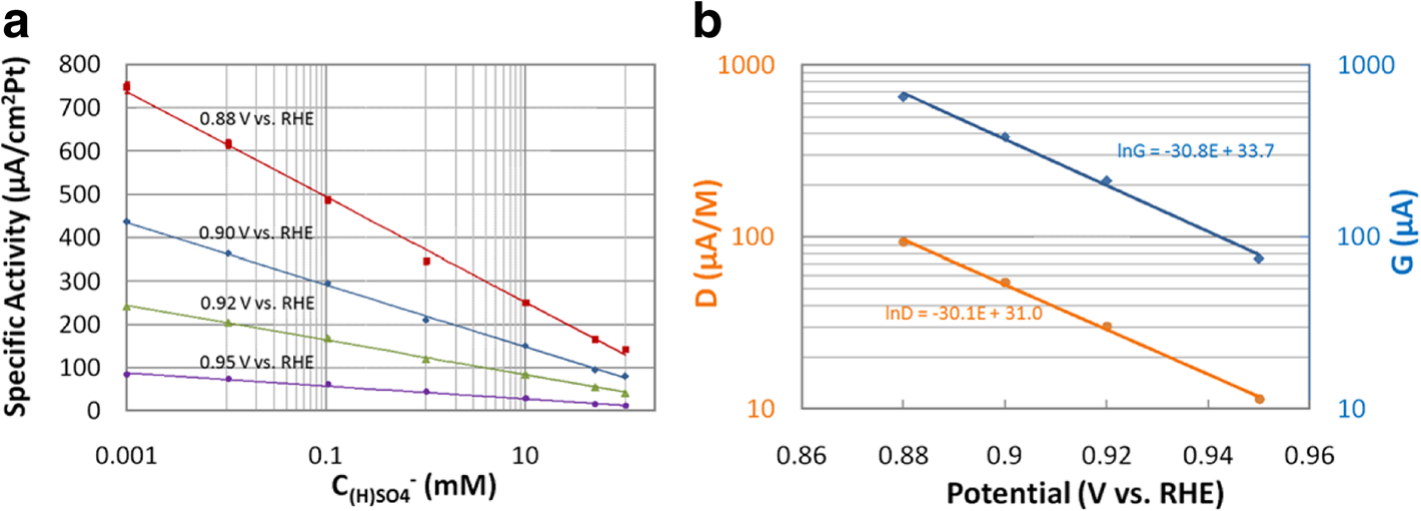
**a** Effect of (bi)sulfate ion concentration on ORR-specific activity at various potentials for 30 wt.% PtCo at a sweep rate of 5 mV/s. **b** Fitted *D* and *G* of Fig. 2a vs. potential

Figure 3a shows ORR-specific activities of 30 wt.% PtCo as a function of (bi)sulfate concentration in 0.1 M HClO_4_. These are averaged repeatable results of two electrodes, and most of the variations between measurements were too small to be visible at this scale. A semilogarithmic linear relationship fits well in the potential range between 0.88 and 0.95 V:

2 $$ I=G-D\ln {C}_{\left(\mathrm{H}\right)\mathrm{S}{\mathrm{O}}_4} $$

ORR activities at 0.9 V in zero (bi)sulfate concentration (521 μA/cm^2^Pt, 0.32 A/mgPt) are consistent with those reported both inside and outside of our laboratory. In addition, the Ag/AgCl reference electrode was connected to the working electrode compartment by a salt bridge. Therefore, the possibility of chloride contamination from the reference electrode could be excluded. The amount of carbon loaded on the glassy carbon disk electrode (35.5 μg/cm^2^) corresponds to ca. six monolayers of carbon. The average thickness of Nafion film (161 μm on the disk, 18.6 μm on the catalyst) is in the magnitude of micrometer. Thus, the thicknesses of carbon and Nafion film are thin enough for the oxygen diffusion. Therefore, the activities we measured should be out of the question.

Using a modified radioactive labeling method, Kolics and Wieckowski [25] established a semilogarithmic (bi)sulfate adsorption isotherm on a Pt(111) electrode:

3 $$ {\theta}_{\left(\mathrm{H}\right)\mathrm{S}{\mathrm{O}}_4}=m\ln {C}_{\left(\mathrm{H}\right)\mathrm{S}{\mathrm{O}}_4}+d $$

where *m* is the slope, and *d* is the intercept of (bi)sulfate ions adsorption isotherm ${\theta}_{\left(\mathrm{H}\right)\mathrm{S}{\mathrm{O}}_4}$ vs. $\ln {\mathrm{C}}_{\left(\mathrm{H}\right)\mathrm{S}{\mathrm{O}}_4}$. If such a semilogarithmic adsorption isotherm is also valid for (bi)sulfate ions on PtCo nanoparticles, the ORR kinetic equation becomes:

4 $$ {\displaystyle \begin{array}{c}I=G+\frac{D}{m}d-\frac{D}{m}\ {\theta}_{\left(\mathrm{H}\right){\mathrm{SO}}_4}\\ {}={nFA}_{\mathrm{Pt}\left(\theta =0\right)}{kC}_{O_2}^{\gamma }{e}^{\left(-\alpha f\eta \right)}\left(1-{\theta}_{\mathrm{oxide}}\right)-{nFA}_{\mathrm{Pt}\left(\theta =0\right)}{kC}_{O_2}^{\gamma }{e}^{\left(-\alpha f\eta \right)}{\theta}_{\left(\mathrm{H}\right){\mathrm{SO}}_4}\\ {}={nFA}_{\mathrm{Pt}\left(\theta =0\right)}{kC}_{O_2}^{\gamma }{e}^{\left(-\alpha f\eta \right)}\left(1-{\theta}_{\mathrm{oxide}}-{\theta}_{\left(\mathrm{H}\right){\mathrm{SO}}_4}\right)\end{array}} $$

where *n* is the number of electrons, *F* is Faradaic constant, *f = F/RT*, *A*_Pt(*θ* = 0)_ is the real initial surface area of catalyst free of adsorbed (bi)sulfate ions and oxide, *k* is the rate constant, ${C}_{{\mathrm{O}}_2}$ is the saturated O_2_ concentration in the electrolyte, *γ* is the reaction order in terms of O_2_ concentration, *θ*_oxide_ and ${\theta}_{\left(\mathrm{H}\right)\mathrm{S}{\mathrm{O}}_4}$ are the fractions of catalyst surface occupied by oxide and (bi)sulfate ions, respectively, *α* is the transfer coefficient, and *η* is the overpotential of the ORR (=*E* − *E*^∗^).

**Fig. 4:**
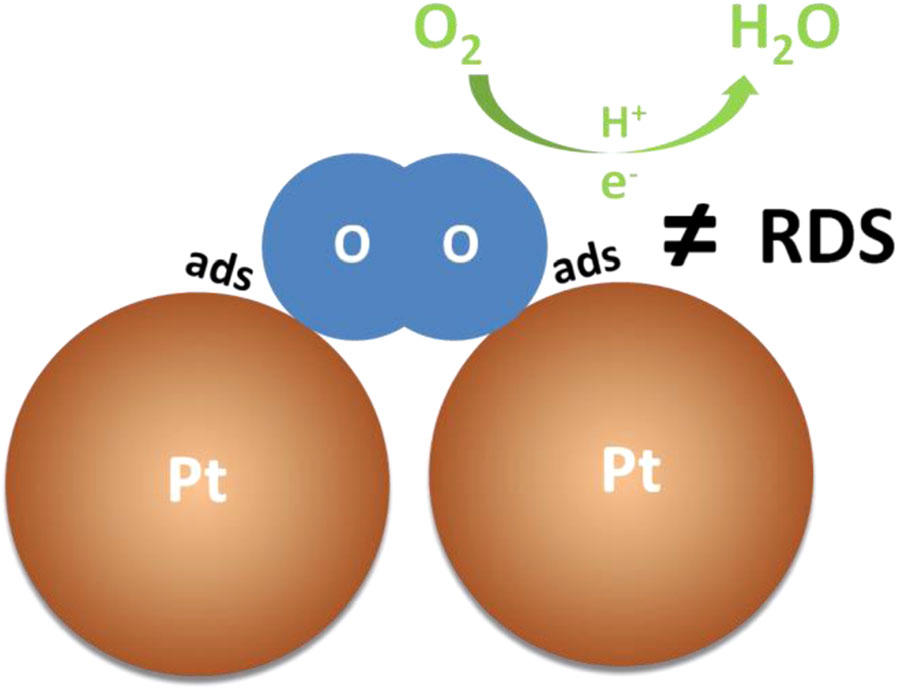
Illustration of ORR mechanism

Equation (4) demonstrates that the exponential term of coverage in the general form of ORR kinetic equation in Reference [31] is not relevant to the effect of (bi)sulfate ion adsorption on ORR catalysis. In other words, (bi)sulfate ion adsorption does not affect the free energy of activation of ORR under a given potential. The reaction order of ORR in terms of available Pt sites is shown to be 1 from Eq. (4), suggesting that these two conditions, the adsorption of one O_2_ molecule onto two Pt sites and this adsorption as a rate-determining step of ORR reaction, are unlikely to exist simultaneously (Fig. 4).

If we note the constant ${K}_1= nF{A}_{\mathrm{Pt}\left(\theta =0\right)}k{C}_{O_2}^{\gamma }{e}^{\left(\alpha f{E}^{\ast}\right)}$, then Eq. (4) becomes:

5a $$ G={K}_1\bullet {e}^{\left(-\alpha fE\right)}\bullet \left(1-d-{\theta}_{\mathrm{oxide}}\right) $$

5b $$ D={K}_1\bullet m\bullet {e}^{\left(-\alpha fE\right)} $$

It can be seen from Fig. 3a that the magnitude of slope increased with decreasing potential, in qualitative agreement with Eq. (5b). The relationships among slopes and intercepts of Fig. 3a are studied below:

According to Eqs. (5), *G* and *D* must follow these relationships:

6a $$ \ln G=-\alpha fE+\ln {K}_1+\ln \left(1-d-{\theta}_{\mathrm{oxide}}\right) $$

6b $$ \ln D=-\alpha fE+\ln {K}_1+\ln (m) $$

In the presence of (bi)sulfate ions, the adsorption of the oxide is greatly inhibited according to an ORR kinetic study on Pt(111) by Wang et al. [39]. Therefore, the change of *θ*_oxide_ with (bi)sulfate concentration and with potential should be negligible especially at high potentials. The change of *d* is also expected to be negligible at high potentials as is shown on Pt(111) [25]. As $\frac{\partial {\theta}_{\mathrm{oxide}}}{\partial E}\approx 0$ and $\frac{\partial d}{\partial E}\approx 0$ as stated above, *m* is almost a constant at high potentials for pure Pt^16^, and Pt alloys are expected to behave similarly; *K*_1_ is constant; therefore,

7a $$ \frac{\partial \ln G}{\partial E}=-\alpha f+\frac{\partial \ln \left(1-d-{\theta}_{\mathrm{oxide}}\right)}{\partial E}=-\alpha f-\frac{1}{1-d-{\theta}_{\mathrm{oxide}}}\bullet \left(\frac{\partial d}{\partial E}+\frac{\partial {\theta}_{\mathrm{oxide}}}{\partial E}\right)\approx -\alpha f $$

7b $$ \frac{\partial \ln D}{\partial E}\approx -\alpha f $$

Eqs. (7) suggest linear relationships of lnG vs. *E* and lnD vs. *E* with identical slope −*αf*. As shown in Fig. 3b, these conditions are well satisfied, and a transfer coefficient of *α* ~ 0.8 is obtained from both slopes, indicating an asymmetric activation energy barrier for the ORR reaction.

**Fig. 5:**
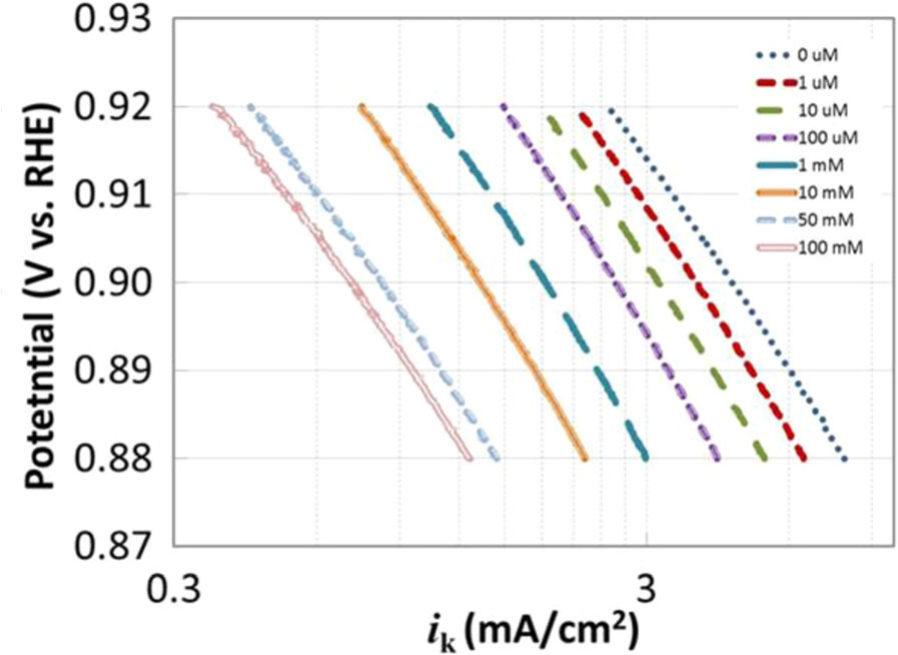
Tafel slope of 30 wt.% PtCo at various (bi)sulfate ion concentrations

Figure 5 shows that the Tafel slope of the ORR reaction is nearly independent of (bi)sulfate concentration, remaining in the range of 77–89 mV/decade. This nearly constant Tafel slope indicates that the mechanistic path of ORR remains independent of the (bi)sulfate adsorption, i.e., (H)SO_4_^−^ anions probably block active Pt sites without changing the rate-determining step of ORR [7, 37].

## Conclusions

The effects of (bi)sulfate poisoning of ORR activities on a PtCo catalyst have been studied at various high potentials. The ORR kinetic current decreases linearly with the logarithm of the anion concentration indicating an ORR kinetic scheme with a transfer coefficient α ~ 0.8. Furthermore, the (bi)sulfate adsorption does not affect the free energy of ORR activation at a given potential. It is unlikely that these two conditions, the adsorption of one O_2_ molecule onto two Pt sites and this adsorption as a rate-determining step of ORR reaction, could exist simultaneously.

## Abbreviations

CV: Cyclic voltammetry
LSV: Linear sweep voltammetry
ORR: Oxygen reduction reaction
PEMFC: Polymer electrolyte membrane fuel cells
RDE: Rotating disk electrode
RHE: Reversible hydrogen electrode
RRDE: Rotating ring-disk electrode
TEM: Transmission electron microscope
XAS: X-ray adsorption spectroscopy
